# Gut microbiota profiles in anorexia nervosa: associations with disease severity, BMI, and history of childhood trauma

**DOI:** 10.3389/fpsyt.2026.1759115

**Published:** 2026-04-15

**Authors:** Meiou Wang, Yu Wang, Jing Ma, Yang Liu, Jin Li, Lan Zhang

**Affiliations:** Mental Health Center, National Center for Mental Disorders, West China Hospital, Sichuan University, Chengdu, China

**Keywords:** eating disorder, anorexia nervosa, childhood trauma, diversity, gut microbiota

## Abstract

**Study objectives:**

Emerging evidence suggests a possible link between anorexia nervosa (AN) and alterations in the gut microbiota. This study aimed to characterize the gut microbiota profile in a cohort of Chinese female patients with AN.

**Method:**

A comparative analysis of the gut microbiota was conducted between 30 female patients with AN and 30 sex- and age-matched healthy controls (HCs). Fecal samples were collected for 16S rRNA gene sequencing analysis. All participants were assessed using the Eating Disorder Inventory (EDI) and the Childhood Trauma Questionnaire (CTQ). Bioinformatics analysis was performed using QIIME2, and statistical analyses were carried out with SPSS 26.0 and R software. Correlations between microbiota differences and body mass index (BMI), EDI, and CTQ were further investigated.

**Results:**

The analysis revealed differences in beta diversity and the abundances of specific microbial taxa between the two groups; however, no significant differences were observed in alpha diversity nor in the associations between gut microbiota and BMI, disease severity, or childhood trauma.

**Conclusions:**

This study identified limited differences in the gut microbiota composition between patients with AN and HCs. Critically, no robust associations between gut microbiota and clinical features were found after rigorous multiple comparison correction. While nominal (uncorrected) correlations were observed between the specific microbiota and psychological traits, these results are exploratory and should be considered hypothesis-generating. They highlight a potential avenue for future research but require validation in larger, longitudinal cohorts to determine their reproducibility and biological significance.

## 1 Introduction

Anorexia nervosa (AN) is a psychiatric disorder characterized by an intense fear of weight gain, significantly low body weight, and a distorted perception of body imag (1). It typically emerges during early adolescence and affects females more frequently than males, with an estimated prevalence of 1.4% (2). AN often results in severe undernutrition and potentially life-threatening medical complications (3), contributing to one of the highest mortality rates among psychiatric illnesses (4). Although the exact etiology and pathophysiology of AN remain incompletely understood, a combination of genetic, developmental, and pre- and post-pubertal biological and psychosocial factors are believed to play a role in its development (5). Nonetheless, these factors alone do not fully explain the complexity of the disorder.

The gut microbiota consists of a complex community of microorganisms, estimated to encompass 10¹³ to 10¹^4^ microbial cells, residing within the human gastrointestinal tract (6). It is predominantly composed of the phyla Bacteroidetes and Firmicutes. Beyond bacteria, the gut microbiota also includes a functionally interconnected network of fungi, viruses, archaea, and eukaryotic microbes (7, 8). This highly interdependent ecosystem plays essential roles in numerous physiological processes, such as regulating tryptophan metabolism (9), synthesizing neuroactive compounds (10), producing neurotransmitters (11), and maintaining gut barrier integrity. Furthermore, it is critically involved in modulating gastrointestinal function, metabolism, immune responses, as well as emotional, cognitive, and behavioral states (12). The composition of the gut microbiota is finely influenced by factors including sex, age, diet, geography, genetics, medication use, and chronic disease (13).

Over the past decade, emerging evidence has implicated dysbiosis of the commensal gut microbiota in mental health disorders, potentially mediated through the gut–brain axis (14). This bidirectional communication network facilitates interaction between the enteric and central nervous systems, thereby influencing mood, cognition, and behavior. Thus, alterations in the gut microbial composition may affect brain processes involved in mood and appetite regulation—functions central to the behavioral manifestations of AN.

Given that AN is characterized by extreme low body weight, and considering the established role of the gut microbiota in energy homeostasis and weight regulation (15), investigating microbial profiles in individuals with AN becomes critically important. Furthermore, gut microbiota composition has been associated with early-life stressors such as childhood trauma (16), which is reported to be more prevalent among AN patients compared to the general population (17). These observations suggest that, particularly in AN patients with a history of childhood trauma, gut microbial alterations may contribute to the pathophysiology of the disorder.

Recent studies have investigated alterations in gut microbiota composition among individuals with AN. Early reports indicated increased abundances of certain archaea such as *Methanobrevibacter smithii* (18–20), which may facilitate energy harvest in a state of chronic caloric restriction (21). However, findings across studies remain inconsistent. Some reports describe reduced microbial diversity (22) and increased abundances of potential pathogens, alongside decreases in beneficial bacteria (23), while others show no significant differences in alpha diversity (24). This heterogeneity indicates that the gut microbiota-AN association is likely subtle and method-dependent. A recent comprehensive synthesis underscores that discrepancies in alpha-diversity findings may be attributed to methodological variations, including differences in stool sample size, analytical techniques, and the choice of diversity indices-each with inherent biases and advantages (25). Consequently, the interpretation of such findings requires caution. These discrepancies highlight the heterogeneity within the field and underscore the need for more standardized, large-scale studies to clarify the role of the gut microbiome in AN.

In summary, current research on the gut microbiota in individuals with AN has identified significant changes in microbial diversity and composition. However, findings have been inconsistent, and data from Chinese populations are notably scarce. Given that women represent approximately 90-95% of the AN-affected population, this study aims to characterize the gut microbiota of Chinese female patients with AN. By comparing the fecal microbiota of these patients with that of age-matched healthy female controls and examining its relationship with BMI, disease severity, and childhood trauma, we seek to advance the understanding of AN’s etiology and pathophysiology and to contribute to the development of future therapeutic strategies.

## 2 Materials and methods

### 2.1 Ethics statement

The study protocol was approved by the Ethics Committee of West China Hospital, Sichuan University (SCU) (2022 Audit No. 1861). Written informed consent was obtained from all adult participants. For participants under 18 years of age, written informed consent was provided by a parent or legal guardian.

### 2.2 Study population and design

A total of 30 female patients with AN were recruited from the outpatient and inpatient services of West China Hospital, Sichuan University, between December 1st, 2021 and December 1st, 2022. Patients were enrolled if they were above 14 years of age and met the DSM-5 diagnostic criteria for AN. Participants were required to have a body mass index (BMI) below 18.5 kg/m², or below the 5th percentile for age and sex in the case of minors. Additionally, eligible participants must not have used any antipsychotics, antidepressants, benzodiazepines, laxatives, diuretics, or other psychotropic or weight-affecting medications within the 8 weeks prior to enrollment. Exclusion criteria included recent use of probiotics, antibiotics, nonsteroidal anti-inflammatory drugs, or steroids; comorbid psychiatric disorders; a history of gastrointestinal surgery (except appendectomy or cholecystectomy); chronic or recurrent bowel-related diagnoses; and current pregnancy or breastfeeding.

During the same period, 30 age-matched healthy female controls were recruited through hospital staff, students, and public advertisements. All potential controls were screened using clinical interviews to confirm the absence of current or past psychiatric disorders. Those selected presented with BMIs within the normal range (18.5-24.9 kg/m²), and the same exclusion criteria applied to the control group as to the patients with AN.

### 2.3 Clinical data collection

For all participants, we collected the demographic data including age, weight, height, and BMI. Stool samples were also obtained from each individual. In addition, trained physicians administered the Eating Disorder Inventory (EDI; Garner, 1983) (26) and the Childhood Trauma Questionnaire (CTQ; Bernstein et al., 1994) (27) to assess all participants.

### 2.4 Psychological assessment

Symptom severity in patients with AN was evaluated via the EDI, a 64-item psychometric instrument assessing the cognitive-affective and behavioral dimensions of eating pathology. The EDI comprises eight validated subscales: Drive for Thinness, Body Dissatisfaction, Bulimia, Perfectionism, Interpersonal Distrust, Interoceptive Awareness, Maturity Fears, and Ineffectiveness. Each item employs a 6-point Likert scale (1 = never to 6 = always), with total scores ranging from 64 to 384; elevated scores correlate with heightened AN symptom severity.

Concurrently, childhood trauma exposure was quantified through the CTQ, a 28-item screening tool encompassing five subdomains: emotional abuse, physical abuse, sexual abuse, emotional neglect, and physical neglect. Responses are recorded on a 5-point frequency scale (1 = never to 5 = always), yielding a composite trauma severity score between 25 and 125 (three items are reserved for validity assessment and are not scored), where higher values indicate greater cumulative trauma burden. It should be noted that retrospective self-report tools like the CTQ capture a specific perspective with inherent limitations, and complementary methods exist for profiling maltreatment dimensions (28). Nevertheless, the CTQ was appropriate for the aims of this study.

### 2.5 Sample collection

Each subject provided a fresh stool sample (approximately 2 g), which was immediately snap-frozen in liquid nitrogen at -80 °C until DNA extraction. Standardized aseptic procedures were followed throughout the collection process.

### 2.6 DNA extraction, amplification, and sequencing

Genomic DNA was extracted from fecal samples using the Omega M5635 Soil DNA Kit (Bio-Tek, USA), and the quality of the extracted DNA was verified by 1.2% agarose gel electrophoresis. The V3–V4 hypervariable regions of the bacterial 16S rRNA gene were amplified using barcoded primer 338F (5’-ACTCCTACGGGGAGGCAGCA-3’) and 806R (5’-GGACTACHVGGGTWTCTAAT-3’). PCR was performed using Pfu high-fidelity DNA polymerase (TransGen Biotech) with minimal cycle numbers to reduce amplification bias. Negative controls were included in all PCR batches. Amplification products were purified using VAHTS™ DNA Clean Beads (Vazyme), quantified with the Quant-iT PicoGreen dsDNA Assay Kit (Thermo Fisher Scientific), and pooled in equimolar ratios based on fluorescence measurements.

Sequencing libraries were constructed using the TruSeq Nano DNA LT Library Prep Kit (Illumina) according to the manufacturer’s instructions, involving end-repair, A-tailing, adapter ligation, and library amplification. Libraries were purified with AMPure XP Beads (Beckman Coulter), and final library quality and size distribution were assessed using an Agilent Bioanalyzer 2100 with the High Sensitivity DNA Kit. Qualified libraries (>2 nM) were denatured and subjected to paired-end sequencing (2 × 300 bp) on an Illumina MiSeq platform using the MiSeq Reagent Kit V3 (600 cycles).

### 2.7 Bioinformatic analysis

Raw paired-end sequencing data were processed using QIIME2 (version 2019.4). First, primers were trimmed using the cutadapt trim-paired method. Subsequently, sequences were denoised, merged, and chimeras were removed using the DADA2 (dada2 denoise-paired). Reads were truncated based on their quality profiles to ensure accurate amplicon sequence variant (ASV) inference. ASVs identified in all samples were merged, and singletons (ASVs with a total count of 1 across all samples) were removed.

For initial community characterization, taxonomic assignment was performed using the Greengenes database (Release 13.8). A pre-trained Naive Bayes classifier within the q2-feature-classifier (classify-sklearn) was employed with default parameters. Any ASVs classified as mitochondria, chloroplasts, archaea, or those unassigned at the kingdom level were filtered out. Prior to diversity analysis, the ASV table was rarefied to standardize sequencing effort across samples. The minimum sequencing depth after quality control was 7, 805 reads per sample. Using qiime feature-table rarefy, the table was subsampled without replacement to 95% of this minimum depth (7, 415 reads per sample). Alpha diversity and beta diversity were calculated based on this Greengenes annotation.

Following recent methodological recommendations, a more robust differential abundance analysis was conducted (29). ASV sequences were re−annotated using the SILVA database (v138.1) with the DADA2 package’s assignTaxonomy function. Differential abundance testing between AN patients and HCs was then performed using ANCOM−BC2 (30), a compositional data analysis method that accounts for sequencing depth and sample heterogeneity while rigorously controlling false discoveries.

### 2.8 Statistical analysis

Statistical analyses were conducted with IBM SPSS Statistics (Version 26.0). The normality of the distribution for continuous variables was assessed using the Shapiro-Wilk test. Continuous variables that were normally distributed are expressed as the mean ± standard deviation (SD) and were compared between groups using the independent-samples t-test. Continuous variables that deviated from a normal distribution are expressed as the median and interquartile range (IQR) and were compared using the Mann-Whitney U test. A *p*-value of < 0.05 was considered statistically significant.

Alpha diversity (including Chao1, observed species, Shannon’s index, Simpson’s index, Faith’s PD, Pielou’s evenness, and Good’s coverage) and beta diversity (Jaccard distance and unweighted UniFrac distance) metrics and species composition analysis were carried out via QIIME2 and R software (Version 4.4.3).

The significance of alpha diversity variation was assessed with the Kruskal-Wallis rank sum test, followed by false discovery rate (FDR) correction using the Benjamini-Hochberg (BH) method for multiple comparisons across the seven indices. Beta diversity variation was assessed using permutational multivariate analysis of variance (PERMANOVA) with 999 permutations. Prior to PERMANOVA, the assumption of homogeneity of multivariate dispersions was tested using the betadisper function in the vegan package (version 2.7-2) to ensure the validity of the results. Taxonomic assignment was performed using the SILVA database (v138.1). Differential abundance analysis between AN patients and HCs was conducted using ANCOM-BC2, a compositional data analysis method that accounts for sequencing depth and sample heterogeneity while controlling the false discovery rate. Significance was defined as a Holm-adjusted *p*-value < 0.05. For comparative purposes, an initial exploratory analysis was also performed using LEfSe with the Greengenes database. In this analysis, statistical significance was determined by the LEfSe statistical tests with FDR correction (*p* < 0.05). The Linear Discriminant Analysis (LDA) score, with a threshold of > 2.0, was used as an estimate of effect size to highlight taxa with larger-magnitude differences between groups. Spearman’s correlations were calculated between the differentially abundant taxa (identified by ANCOM-BC2) and clinical variables, including BMI, EDI subscales, CTQ subscales, and disease duration. P-values were adjusted for multiple testing using the Holm method, with a Holm-adjusted p < 0.05 considered statistically significant for the ANCOM-BC2 analysis.

## 3 Results

### 3.1 Clinical characteristics

Patients with AN exhibited significantly lower body weight and BMI compared to HCs. Furthermore, exploratory comparisons on psychological assessments revealed elevated levels of eating disorder psychopathology and childhood trauma in the AN group (**Table 1**).

**Table 1:** Clinical sample characteristics.

Variable	AN (n = 30)	HC (n = 30)	*t*	*p*-value
Age, (years)	25.43 ± 6.51	26.37 ± 5.14	-0.62	0.540
Height, (cm)	160.87 ± 5.09	160.43 ± 4.56	0.35	0.730
Weight, (kg)	43.40 ± 6.23	54.42 ± 6.18	-6.87	<0.001**
BMI, (kg/m^2^)	16.72 ± 1.99	21.13 ± 1.85	-8.88	<0.001**
EDI (total score)	233.70 ± 32.27	182.47 ± 70.04	3.64	0.001**
EDI drive for thinness	26.20 ± 6.34	16.80 ± 7.71	5.16	<0.001**
EDI body dissatisfaction	26.13 ± 4.79	25.37 ± 10.75	0.36	0.723
EDI bulimia	29.33 ± 8.54	20.00 ± 7.77	4.43	<0.001**
EDI perfectionism	23.47 ± 6.56	17.60 ± 7.30	3.27	0.002**
EDI interpersonal distrust	25.03 ± 4.34	20.40 ± 8.28	2.71	0.009**
EDI maturity fears	27.00 ± 4.63	22.80 ± 9.49	2.18	0.033*
EDI interoceptive	37.83 ± 8.61	29.07 ± 8.61	3.36	0.001**
EDI inefficacy	34.50 ± 6.60	27.87 ± 11.41	2.93	0.005**
CTQ (total score)	46.17 ± 14.19	38.13 ± 6.96	2.78	0.007**
CTQ emotional abuse	9.67 ± 4.04	7.23 ± 2.52	2.80	0.007**
CTQ physical abuse	6.83 ± 2.68	5.70 ± 1.64	1.98	0.053
CTQ sexual abuse	6.07 ± 2.21	5.23 ± 0.77	1.95	0.056
CTQ emotional neglect	14.63 ± 5.40	12.30 ± 2.81	2.10	0.040*
CTQ physical neglect	8.97 ± 3.40	7.67 ± 2.75	1.63	0.110

Values are presented as mean ± SD. AN, anorexia nervosa; HC, healthy control; BMI, body mass index; EDI, Eating Disorder Inventory; CTQ, Childhood Trauma Questionnaire. Statistical comparisons of individual subscales are descriptive and exploratory, as p-values are uncorrected for multiple testing. t was for the T-test. *p < 0.05. **p < 0.01.

### 3.2 Microbiome composition and diversity

Initial sequencing of the gut microbiota yielded 1, 299, 711 raw sequences. After quality filtering, chimera removal, and pair-end merging, we obtained 705, 083 high-quality sequences. The length distribution of these merged sequences peaked between 400 to 430 base pairs (**Supplementary Figure 1a**). A total of 15, 540 ASVs were identified via species taxonomic annotation, providing comprehensive insights into the microbial diversity of the sample population (**Supplementary Figure 1b**). The plateauing of the rarefaction curves suggested adequate sequencing depth for robust analysis of our datasets (**Supplementary Figure 1c**).

**Figure 1:**
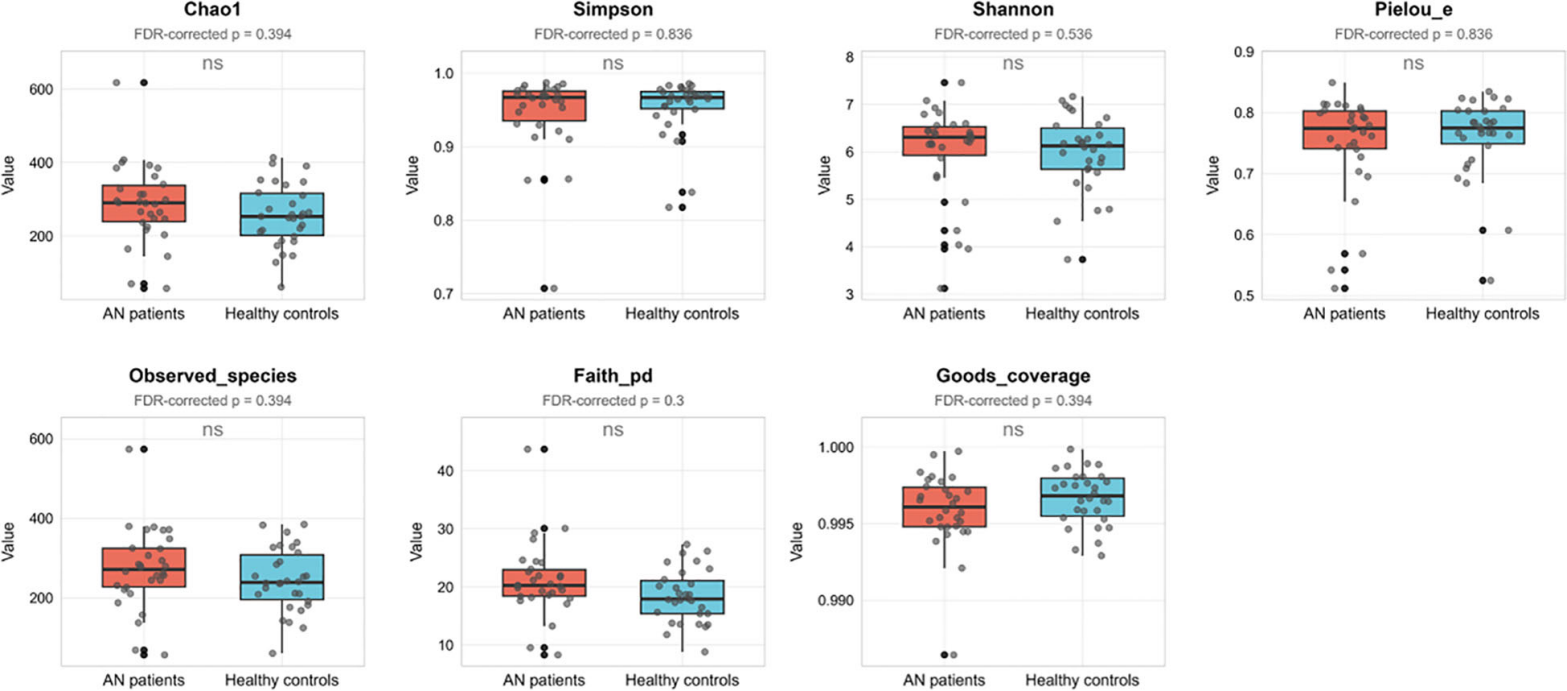
Comparison of gut microbial alpha diversity between AN patients and HCs. Boxplots display seven alpha diversity indices. Boxes show median and IQR, points are individual samples. Statistical comparisons were performed using the Kruskal-Wallis rank sum test, with p-values adjusted for multiple testing using the FDR method. No significant inter-group differences were observed (all adjusted p > 0.05). AN, anorexia nervosa; HCs, healthy controls; IQR, interquartile range.

Comparison using the Kruskal-Wallis test with FDR correction revealed no significant differences in alpha diversity between AN patients and HCs (all FDR-corrected p > 0.05, see **Figure 1**; for uncorrected and corrected *p*-values, see **Supplementary Table 1**). In contrast, permutational multivariate analysis of variance (PERMANOVA) revealed a statistically significant but weak separation in overall community structure between patients with AN and HCs (Jaccard distance: R² = 0.019, *p* = 0.043; unweighted UniFrac distance: R² = 0.026, *p* = 0.019). A test for homogeneity of multivariate dispersions (Betadisper) confirmed no significant difference in dispersion between groups (Jaccard: F = 0.451, *p* = 0.505; unweighted UniFrac: F = 1.420, *p* = 0.238), validating the PERMANOVA model assumptions. This separation was visualized using non-metric multidimensional scaling (NMDS) ordination (**Supplementary Figures 2A, B**, **Table 2**).

**Table 2:** Beta diversity difference test in in AN patients and HCs.

Distance	Sums of sqs	Mean sqs	F.model	R^2^	*p*-value
Jaccard	0.464	0.464	1.126	0.019	0.043*
Unweighted UniFrac	0.296	0.296	1.533	0.026	0.019*

Beta-diversity was compared using Permutational Multivariate Analysis of Variance (PERMANOVA) with 999 permutations. The assumption of homogeneity of multivariate dispersions was confirmed prior to analysis (betadisper test, all *p* > 0.05). The analysis was performed on Jaccard (qualitative) and unweighted UniFrac (phylogenetic) distance matrices. AN, anorexia nervosa; HC, healthy control. * *p* < 0.05.

**Figure 2:**
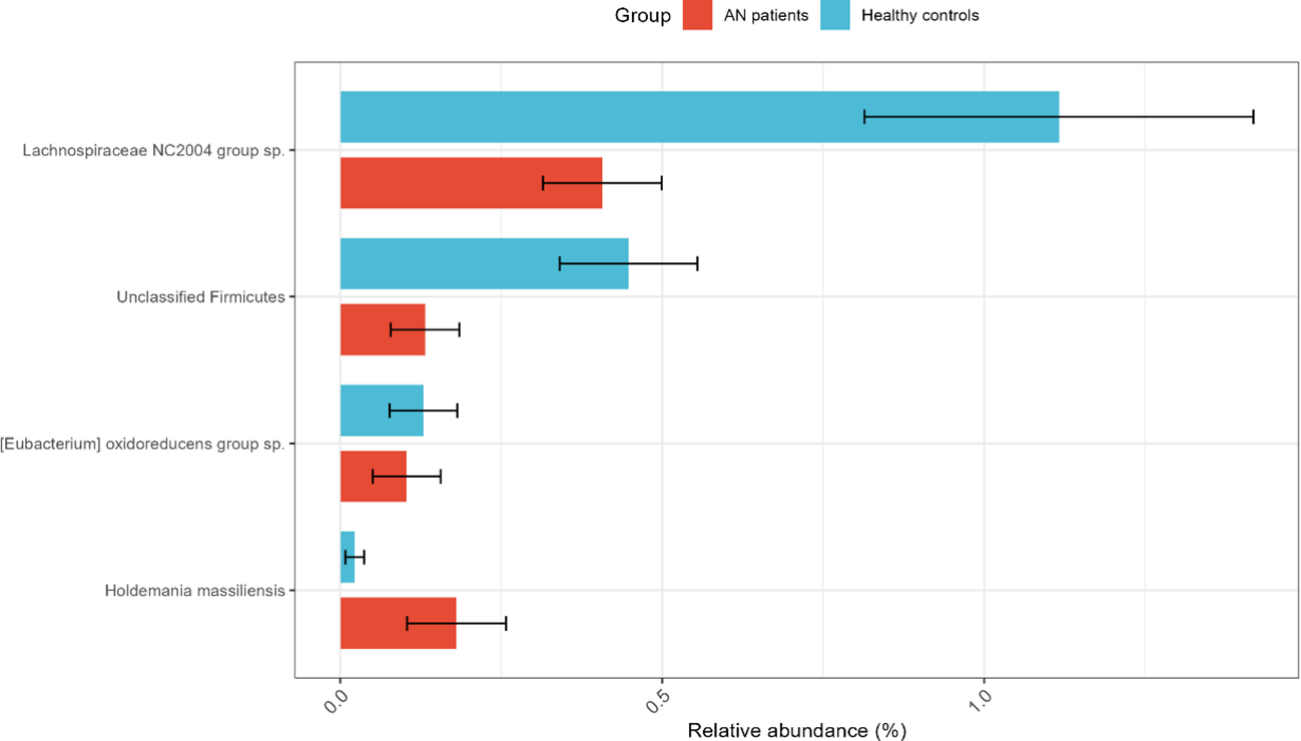
Differentially abundant taxa with prevalence ≥20%. Bars represent mean relative abundance (%) ± SE. Three taxa, *Lachnospiraceae NC2004 group* sp., an unclassified Firmicutes, and [*Eubacterium*] *oxidoreducens group* sp., were more abundant in healthy controls, while *Holdemania massiliensis* was more abundant in AN patients (FDR < 0.05, ANCOM-BC2). Full statistics for all 12 significant taxa are provided in **Table 3**.

Differential abundance analysis using ANCOM-BC2 with the SILVA database (v138.1) identified 12 taxa significantly different between AN patients and HCs (Holm-adjusted p < 0.05; **Table 3**). To highlight robust signals, the four taxa with prevalence ≥20% are visualized in **Figure 2**. Among these, *Lachnospiraceae NC2004 group* sp., an unclassified Firmicutes, and [*Eubacterium*] *oxidoreducens group* sp. were more abundant in healthy controls, whereas *Holdemania massiliensis* was more abundant in AN patients. The remaining eight taxa (prevalence < 20%)-including members of *Faecalibacterium*, *Monoglobus*, *CHKC1002*, *Dorea*, *Erysipelatoclostridium*, and unclassified Actinobacteria-are detailed in **Table 3**. For comparative purposes, an initial exploratory analysis using LEfSe with the Greengenes database is provided in **Supplementary Table 2**, **Supplementary Figure 3**.

**Table 3:** Differentially abundant taxa between anorexia nervosa (AN) and healthy control (HC) groups identified by ANCOM-BC2.

ASV ID	Taxon	Phylum	Family	Genus	Species	Log2FC (AN/HC)	*p*-value	Adjusted *p*-value
ASV_3530	Unclassified Firmicutes	Firmicutes	Lachnospiraceae	NA	NA	-1.66	<0.001	0.00118
ASV_8383	Dorea sp.	Firmicutes	Lachnospiraceae	Dorea	NA	1.34	<0.001	0.00464
ASV_4413	Faecalibacterium sp.	Firmicutes	Ruminococcaceae	Faecalibacterium	NA	-1.48	<0.001	0.00679
ASV_2818	Lachnospiraceae NC2004 group sp.	Firmicutes	Lachnospiraceae	Lachnospiraceae NC2004 group	NA	-1.12	<0.001	0.01402
ASV_8710	Holdemania massiliensis	Firmicutes	Erysipelotrichaceae	Holdemania	massiliensis	1.2	<0.001	0.01604
ASV_6319	Unclassified Actinobacteriota	Actinobacteriota	NA	NA	NA	-2.4	<0.001	0.01851
ASV_5278	Erysipelatoclostridium sp.	Firmicutes	Erysipelatoclostridiaceae	Erysipelatoclostridium	NA	2.18	<0.001	0.01957
ASV_1620	Lachnospiraceae NC2004 group sp.1	Firmicutes	Lachnospiraceae	Lachnospiraceae NC2004 group 1	NA	1.38	<0.001	0.02571
ASV_2573	Unclassified Firmicutes	Firmicutes	Lachnospiraceae	NA	NA	-0.81	<0.001	0.03422
ASV_4833	Monoglobus sp.	Firmicutes	Monoglobaceae	Monoglobus	NA	-1.04	<0.001	0.03446
ASV_1385	CHKCI002 sp.	Actinobacteriota	Eggerthellaceae	CHKCI002	NA	1.24	<0.001	0.0409
ASV_7879	[Eubacterium] oxidoreducens group sp.	Firmicutes	Lachnospiraceae	[Eubacterium] oxidoreducens group	NA	-1.1	<0.001	0.04328

Taxa with adjusted *p*-value < 0.05 (ANCOM−BC2) are shown. ASV, amplicon sequence variant. Log2 fold change (Log2FC) represents the difference in abundance between AN and HC groups; positive values indicate higher abundance in the AN group. Taxonomy was assigned using the SILVA database (v138.1). For each taxon, the finest available taxonomic classification is provided. Adjusted p-values were calculated using the Holm method for multiple testing correction.

### 3.3 Correlations among gut microbiota, BMI, disease severity, and childhood trauma

Spearman correlation analysis was conducted between the 12 taxa identified as significantly differentially abundant by ANCOM-BC2 (Holm-adjusted p < 0.05) and clinical variables, including eating disorder severity (EDI subscales), childhood trauma (CTQ subscales), and BMI. *P*-values were corrected for multiple comparisons using the FDR. After FDR correction, no taxon-clinical variable pair remained statistically significant (all q > 0.05). To visualize the overall correlation structure, a bubble plot of all Spearman coefficients is presented in **Supplementary Figure 4**, where circle size reflects the absolute correlation coefficient and color indicates the direction (red: positive, blue: negative). To further illustrate potential trends, the 10 strongest correlations (by absolute coefficient) are summarized in **Supplementary Table 4**, together with their nominal and FDR-corrected *p*-values. These nominal associations should be interpreted as hypothesis-generating only, as they did not survive correction. For completeness, an initial broader correlation analysis using all taxa with |r| > 0.3 from the LEfSe-based exploration is provided in **Supplementary Table 3**, which includes nominal and FDR−corrected p-values for all such correlations from the initial exploratory analysis.

## 4 Discussion

This study employed 16S rRNA sequencing to characterize the fecal microbiota of Chinese female patients with AN and to investigate its potential associations with BMI, disease severity, and childhood trauma. Our results revealed distinct differences in gut microbial diversity and composition between AN patients and HCs.

Our findings indicated no significant differences in alpha diversity between patients with AN and HCs, which is consistent with reports by Yuan et al. (24) and Andreani et al. (31). In contrast, several other studies—such as those by Hanachi et al. (23) and Kleiman et al. (32) - described reduced alpha diversity in participants with restrictive AN. Similarly, Monteleone et al. (22) observed decreased alpha diversity in restrictive AN patients but not in those with binge-purging AN, while Mack et al. (19) and Borgo et al. (20) reported significant alpha diversity differences between patients with AN and controls. These discrepancies across studies may be attributable to variations in sample size, subtype, severity, or chronic dietary habits. Notably, the fact that approximately 90% of the patients in our cohort were diagnosed with the binge-purging subtype of AN may also account for the lack of significant alpha diversity differences observed.

Consistent with our findings, significant differences in beta diversity were also reported in previous studies by Hanachi et al. (23) and Morkl et al. (33). Although we observed a statistically significant difference, the effect size was notably small (R² < 0.03). It indicates that AN diagnosis explains less than 3% of the total variance in gut microbiota composition. The majority of microbial variation is likely attributable to other factors such as inter-individual variation, habitual diet, physical activity levels, and environmental exposures. Consequently, this shift should be interpreted as a subtle community-level change superimposed on a background of high interpersonal variability, rather than a dominant, disease-defining feature.

A key finding of this study was the differential abundance of multiple taxa within the Lachnospiraceae family between AN patients and HCs, consistent with previous reports by Yuan et al. who also observed enrichment of Lachnospiraceae in AN (24). Notably, our use of the SILVA database enabled finer taxonomic resolution, revealing a bidirectional pattern within this family: while some members (e.g., unclassified Lachnospiraceae and members of the [Eubacterium] oxidoreducens group) were decreased in AN, others (e.g., Dorea sp.) were increased in AN. This complexity may have been masked in earlier studies using lower-resolution databases. The potential clinical relevance of Lachnospiraceae in AN is further supported by a recent Mendelian randomization study by Yu et al., which identified a causal relationship between the Lachnospiraceae NC2004 group and AN risk (IVW OR = 1.287, 95% CI 1.019-1.626, *p* = 0.034) (34). Our observation of decreased abundance of this same taxon in AN provides complementary evidence from a direct case-control comparison, strengthening the case for its involvement in AN pathophysiology.

Schulz et al. have proposed that Lachnospiraceae, particularly unclassified members, may serve as predictors of clinical outcome in AN, with increased abundance at admission associated with shorter inpatient treatment duration (35). Mechanistically, Lachnospiraceae are known to degrade otherwise indigestible dietary components, potentially enhancing energy extraction- a function of particular relevance in undernourished AN patients. Their capacity to produce butyrate, a short-chain fatty acid crucial for intestinal barrier integrity and limiting colonic inflammation, also aligns with the low-grade inflammation observed in AN (36). Thus, the bidirectional changes observed in our study may reflect distinct functional roles of different Lachnospiraceae members, with some potentially contributing to energy harvest and anti-inflammatory effects (those enriched in HC), while others may respond differently to the nutritional and physiological stressors characteristic of AN (those enriched in AN). Future studies integrating metagenomic sequencing, metabolomic profiling, and longitudinal clinical data are warranted to elucidate the functional consequences of these compositional shifts and to explore whether specific Lachnospiraceae taxa could serve as therapeutic targets or prognostic biomarkers in AN.

Our exploratory correlation analysis revealed nominal associations between certain gut microbial taxa and specific psychological traits in AN, such as BMI, bulimia, sexual abuse, emotional neglect and interoceptive awareness. It is critical to emphasize that these associations did not survive correction for multiple comparisons and must therefore be interpreted strictly as preliminary and hypothesis-generating. Our findings highlight potential microbiota-psychology links that warrant targeted validation in future studies but do not establish causality.

The absence of robust, direct associations between the gut microbiota and CTQ severity in our cross-sectional study aligns with a growing understanding that early adversity often influences long-term health outcomes indirectly. Specifically, its effects on physiological systems, including potentially the gut ecosystem, may be mediated by intervening psychological and social constructs, structured style values, coping styles, or social competence, which were not measured in our design. This indirect pathway hypothesis is supported by models in psychopathology, where childhood stress has been shown to shape adult mental health outcomes primarily through its impact on such mediating resources, rather than through direct effects. Therefore, our null finding regarding a direct microbial signature of trauma does not preclude a role for early adversity in AN, but suggests that its influence is likely embedded within more complex, psychologically mediated pathways that future research should aim to elucidate (37).

It is important to acknowledge that among the 12 taxa identified as differentially abundant, several exhibited low prevalence (present in < 20% of samples in either group). While statistically significant after multiple testing correction, the biological relevance of such rare taxa must be interpreted with caution. Their sporadic detection may reflect inter-individual variability, sampling depth limitations, or true low-abundance members that could nonetheless play context-dependent roles. However, given their rarity, these taxa are unlikely to represent core microbial features consistently associated with AN, and their potential functional contributions remain speculative. Future studies with larger cohorts and deeper sequencing are warranted to validate these findings and to determine whether these rare taxa represent meaningful biological signals or stochastic noise.

Several limitations of this study should be acknowledged. First, detailed clinical characterization of the AN cohort was limited. Variables such as treatment setting (inpatient/outpatient), stage of treatment and refeeding, recent weight change, gastrointestinal symptoms, bowel habits, smoking, menstrual status, and supplement use were not systematically collected. This constrains our ability to attribute observed differences specifically to the disease pathophysiology rather than to its treatment or acute manifestations. Second, other potential confounders such as dietary patterns were not controlled. Third, the absence of biomarkers assessing intestinal permeability or systemic inflammation limits functional interpretation of the observed microbiota changes. Fourth, the relatively small sample size necessitates validation in a larger cohort to enhance the robustness of our findings. Fifth, as this study focused exclusively on Chinese female participants, the generalizability of the results to other populations may be limited. Sixth, as an exploratory study focused on differential abundance, we did not perform a core microbiome analysis. Future studies with larger cohorts could investigate the core microbiota to complement our findings. Seventh, because our study was designed to investigate bacterial communities using 16S rRNA sequencing, we did not assess archaeal taxa such as *Methanobrevibacter*, whose potential role in AN remains to be explored in future studies. Finally, the cross-sectional design precludes causal inference. Future longitudinal studies incorporating pre- and post-treatment microbiota profiling would help clarify the nature and dynamics of gut microbiome alterations in AN.

## 5 Conclusions

This exploratory study of Chinese females with AN revealed differences in gut microbial beta diversity and composition compared to HCs. However, after rigorous correction for multiple comparisons, no significant associations were found between gut microbiota and key clinical measures (e.g., BMI, eating disorder psychopathology). Nominal (uncorrected) correlations between the specific microbiota and psychological traits are considered hypothesis-generating and require independent validation. These results provide an initial characterization of the gut microbiome in this population and highlight the need for further investigation in larger, longitudinal studies to determine their biological relevance to AN.

## Data Availability

The raw 16S rRNA gene sequencing data supporting this study are deposited in the NCBI Sequence Read Archive (SRA) under the BioProject accession number PRJNA1427629.
