# Supplementary material for: Gut microbiota profiles in anorexia nervosa: associations with disease severity, BMI, and history of childhood trauma
Source: Front Psychiatry. 2026 Apr 15;17:1759115. doi: 10.3389/fpsyt.2026.1759115 (PMC13125984; doi:10.3389/fpsyt.2026.1759115)
Supplement: Supplementary file 1 [file DataSheet1.pdf]

Supplementary Materials Online

Gut Microbiota Profiles in Anorexia Nervosa: Associations with Disease Severity, BMI, and History of Childhood Trauma

Meiou Wang [a], Yu Wang [a], Jing Ma [a], Yang Liu [a], Jin Li [a], Lan Zhang [a,*]

Affiliation:

[a] Mental Health Center, National Center for Mental Disorders, West China Hospital, Sichuan University, Chengdu, China

Corresponding author:

Lan Zhang,

Mental Health Center, National Center for Mental Disorders, West China Hospital, Sichuan University, Dian Xin Nan Jie 28#, Chengdu 610041, China.

Email address: huaxizhanglan@126.com

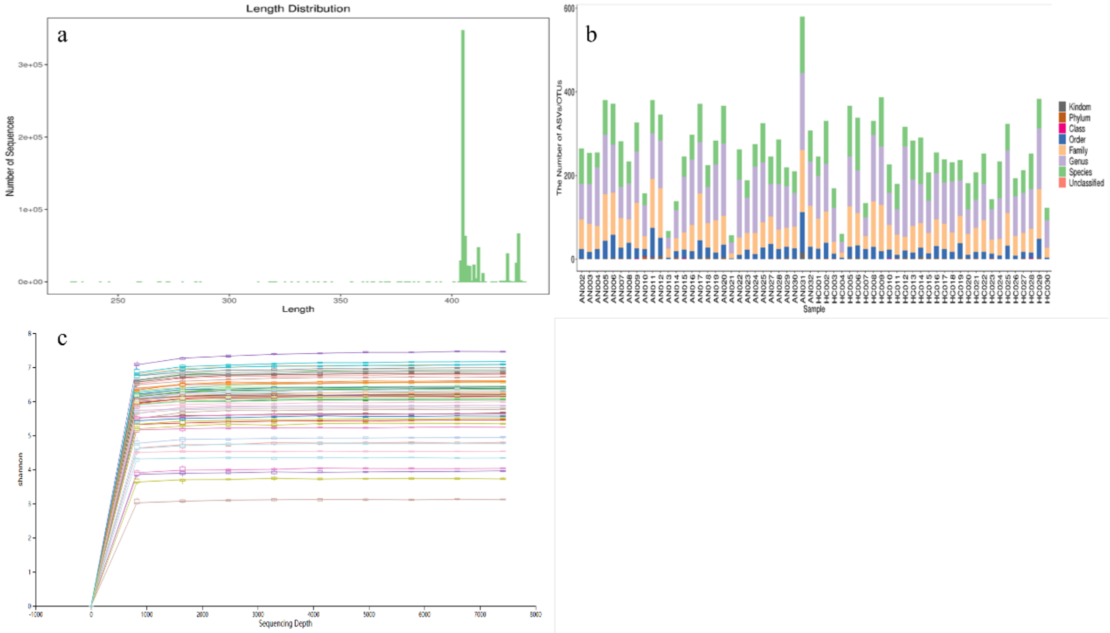

Figure S1. Sequence length distribution map (a); Statistical chart of taxonomic annotation results (b); Sparse curve of each sample (c).

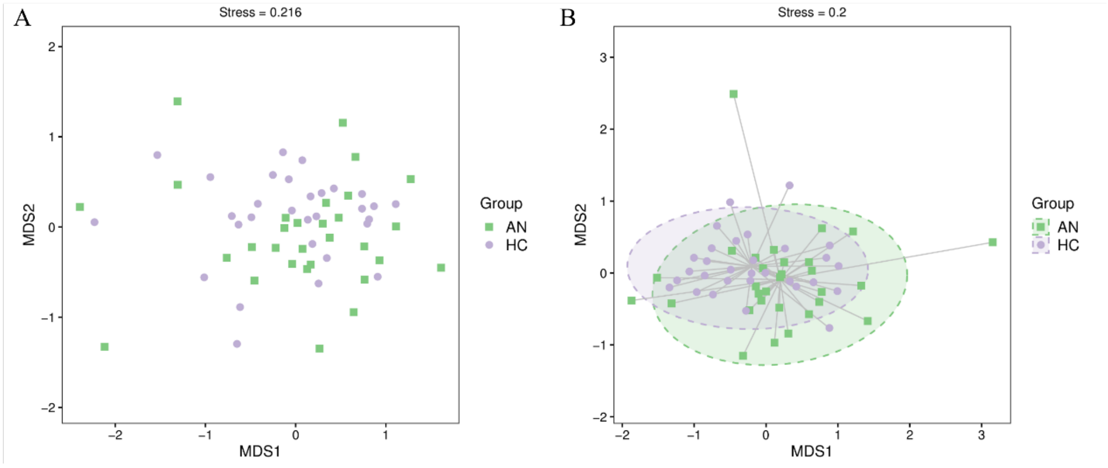

Figure S2. Beta diversity analysis of gut microbial communities. Non-metric multidimensional scaling (NMDS) ordination plots based on (A) Jaccard distance and (B) unweighted UniFrac distance, comparing patients with AN and HCs. The stress values (0.216 for A, 0.20 for B) indicate an acceptable though limited representation of the multidimensional data. The visual separation between groups is subtle, which aligns with the low explanatory power (PERMANOVA $R^2 = 0.019$ for A, 0.026 for B) of the group factor in the corresponding statistical test. AN, anorexia nervosa; HC, healthy control.

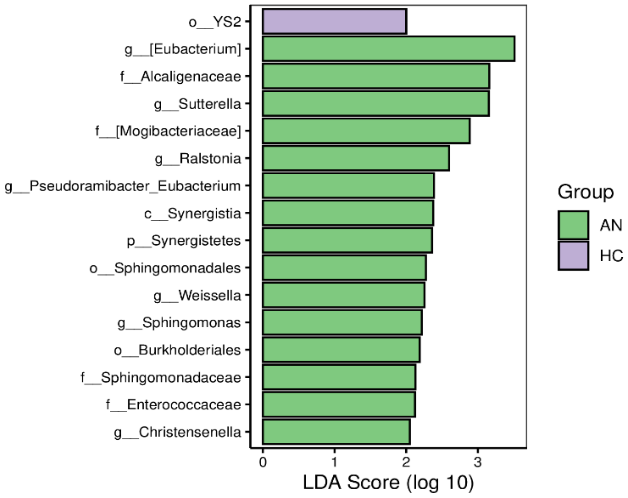

Figure S3. Effect sizes of differentially abundant microbial taxa. Histogram showing the Linear Discriminant Analysis (LDA) scores for microbial taxa identified by LEfSe analysis. Statistical significance was determined by the LEfSe statistical tests (FDR-corrected $p < 0.05$). The LDA score reflects the magnitude of difference (effect size) in relative abundance between patients with AN and HCs. Only taxa with an LDA score > 2.0 are displayed. HCs, healthy controls; AN, anorexia nervosa; LEfSe, linear discriminant effects; LDA, linear discriminant analysis; p, Phylum; c, Class; o, Order; f, Family; g, Genus.

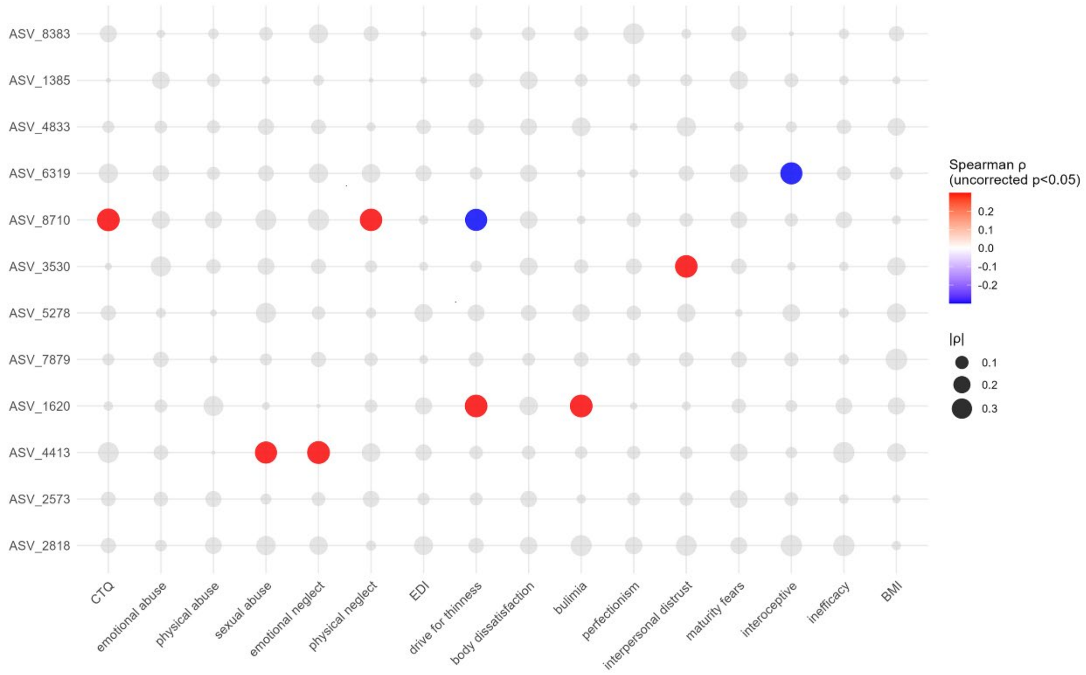

Figure S4. Bubble plot of Spearman correlations between clinical variables and 12 differentially abundant taxa.

Note: Each correlation is represented by a circle. Circle size reflects the absolute correlation coefficient (|ρ|). Gray circles indicate correlations with uncorrected $p \geq 0.05$; colored circles highlight correlations with uncorrected $p < 0.05$ (blue: negative, red: positive). After Benjamini-Hochberg FDR correction for multiple testing, no correlations were statistically significant (all adjusted $p > 0.05$); therefore, all displayed associations are descriptive and should be interpreted with caution. The color scale is fixed at -0.3 to 0.3 for consistency with common correlation benchmarks. The ten strongest correlations are listed in Supplementary Table S4.
