# Supplementary material for: Gut microbiota profiles in anorexia nervosa: associations with disease severity, BMI, and history of childhood trauma
Source: Front Psychiatry. 2026 Apr 15;17:1759115. doi: 10.3389/fpsyt.2026.1759115 (PMC13125984; doi:10.3389/fpsyt.2026.1759115)
Supplement: Supplementary file 2 [file Table1.docx]

**Supplementary Materials Online**

**Gut Microbiota Profiles in Anorexia Nervosa: Associations with Disease Severity, BMI, and History of Childhood Trauma**

Meiou Wang ^a^, Yu Wang ^a^, Jing Ma ^a^, Yang Liu ^a^, Jin Li ^a^, Lan Zhang ^a,*^

**Affiliation:**

^a^ Mental Health Center, National Center for Mental Disorders, West China Hospital, Sichuan University, Chengdu, China

**Corresponding author:**

Lan Zhang,

Mental Health Center, National Center for Mental Disorders, West China Hospital, Sichuan University, Dian Xin Nan Jie 28#, Chengdu 610041, China.

**Email address:** huaxizhanglan@126.com

**Table S1**. Alpha diversity in AN patients and HCs.

| Index | AN (n = 30)  Median (IQR) | HC (n = 30)  Median (IQR) | H | *p*-value | FDR-Corrected *p* |
| --- | --- | --- | --- | --- | --- |
| Chao1 | 289.99(233.71,346.01) | 253.30(195.56,323,07) | 1.770 | 0.183 | 0.394 |
| Simpson | 0.97(0.93,0.98) | 0.97(0.95,0.98) | 0.043 | 0.846 | 0.836 |
| Shannon | 6.31(5.78,6.56) | 6.13(5.61,6.55) | 0.761 | 0.383 | 0.536 |
| Pielou_e | 0.77(0.74,0.80) | 0.77(0.74,0.80) | 0.087 | 0.767 | 0.836 |
| Observed_species | 271.40(225.13,330.73) | 238.90(189.05,317.20) | 1.810 | 0.179 | 0.394 |
| Faith_pd | 20.26(18.33,23.35) | 17.92(14.98,21.55) | 4.103 | 0.043* | 0.301 |
| Goods_coverage | 1.00(0.99,1.00) | 1.00(1.00,1.00) | 1.470 | 0.225 | 0.394 |

Note: Values are presented as median (IQR); AN, anorexia nervosa; HC, Healthy control; H was for the Kruskal-Wallis test; all p-values were corrected for multiple comparisons using the false discovery rate (FDR) method, with a significance threshold of 0.05. **p* < 0.05.

**Table S2.** Differentially abundant microbial taxa between AN patients and HCs

| Taxa | Relative abundance | LDA | *p*-value | FDR-Corrected *p* |
| --- | --- | --- | --- | --- |
| *Eubacterium* | 4.076 | 3.512 | 0.019 | 0.04 |
| Alcaligenaceae | 3.563 | 3.160 | 0.028 | 0.04 |
| *Sutterella* | 3.563 | 3.153 | 0.028 | 0.04 |
| Mogibacteriaceae | 3.344 | 2.886 | 0.027 | 0.04 |
| *Ralstonia* | 2.991 | 2.599 | 0.021 | 0.04 |
| *Pseudoramibacter-*  *Eubacterium* | 2.499 | 2.388 | 0.005 | 0.04 |
| Synergistia | 1.852 | 2.377 | 0.021 | 0.04 |
| Synergistetes | 1.852 | 2.361 | 0.021 | 0.04 |
| Sphingomonadales | 2.028 | 2.276 | 0.040 | 0.04 |
| *Weissella* | 2.550 | 2.254 | 0.036 | 0.04 |
| *Sphingomonas* | 2.028 | 2.218 | 0.040 | 0.04 |
| Burkholderiales | 1.661 | 2.188 | 0.040 | 0.04 |
| Sphingomonadaceae | 2.028 | 2.130 | 0.040 | 0.04 |
| Enterococcaceae | 2.102 | 2.124 | 0.040 | 0.04 |
| *Christensenella* | 2.259 | 2.052 | 0.021 | 0.04 |
| YS2^†^ | 1.845 | 2.001 | 0.040 | 0.04 |

Note: Microbial taxa identified with significant differential abundance between AN patients and HCs by LEfSe analysis (FDR-corrected p < 0.05). All listed taxa, except where noted, were enriched in the AN group. † Taxon YS2 showed higher relative abundance in the HC group. Statistical significance is determined by the FDR-corrected p-value. LDA score reflects the magnitude (effect size) of the abundance difference between groups, with LDA > 2.0 indicating larger effects.

**Table S3**. Correlation between microbial abundance and clinical variables in patients with AN

| Microbe | Variable | Correlation | *p*-value | FDR-Corrected *p* |
| --- | --- | --- | --- | --- |
| Synergistetes | physical abuse | 0.336 | 0.069 | 0.776 |
| Synergistia | physical abuse | 0.336 | 0.069 | 0.776 |
| Mogibacteriaceae | physical abuse | 0.347 | 0.061 | 0.776 |
| *Eubacterium* | physical abuse | 0.359 | 0.051 | 0.776 |
| Mogibacteriaceae | sexual abuse | -0.339 | 0.067 | 0.776 |
| Sphingomonadaceae | EDI | -0.324 | 0.080 | 0.776 |
| *Pseudoramibacter_Eubacterium* | EDI | -0.355 | 0.054 | 0.776 |
| *Sphingomonas* | EDI | -0.324 | 0.080 | 0.776 |
| Synergistetes | drive for thinness | -0.329 | 0.076 | 0.776 |
| Synergistia | drive for thinness | -0.329 | 0.076 | 0.776 |
| Sphingomonadales | drive for thinness | -0.304 | 0.102 | 0.776 |
| Burkholderiales | drive for thinness | -0.440 | 0.015 | 0.776 |
| Sphingomonadaceae | drive for thinness | -0.304 | 0.102 | 0.776 |
| *Ralstonia* | drive for thinness | -0.393 | 0.031 | 0.776 |
| *Pseudoramibacter_Eubacterium* | drive for thinness | -0.516 | 0.004 | 0.776 |
| *Sphingomonas* | drive for thinness | -0.304 | 0.102 | 0.776 |
| Sphingomonadales | bulimia | -0.331 | 0.074 | 0.776 |
| Sphingomonadaceae | bulimia | -0.331 | 0.074 | 0.776 |
| *Pseudoramibacter_Eubacterium* | bulimia | -0.333 | 0.072 | 0.776 |
| *Sphingomonas* | bulimia | -0.331 | 0.074 | 0.776 |
| Enterococcaceae | perfectionism | 0.303 | 0.103 | 0.776 |
| Sphingomonadales | interpersonal distrust | -0.404 | 0.027 | 0.776 |
| Sphingomonadaceae | interpersonal distrust | -0.404 | 0.027 | 0.776 |
| *Eubacterium* | interpersonal distrust | -0.461 | 0.010 | 0.776 |
| *Sphingomonas* | interpersonal distrust | -0.404 | 0.027 | 0.776 |
| Mogibacteriaceae | inefficacy | -0.319 | 0.086 | 0.776 |
| *Pseudoramibacter_Eubacterium* | inefficacy | -0.312 | 0.093 | 0.776 |
| Synergistetes | BMI | 0.314 | 0.091 | 0.776 |
| Synergistia | BMI | 0.314 | 0.091 | 0.776 |
| Burkholderiales | BMI | 0.323 | 0.081 | 0.776 |
| Burkholderiales | Duration | 0.390 | 0.033 | 0.776 |
| *Ralstonia* | Duration | 0.346 | 0.061 | 0.776 |

Note: Statistical significance is determined by the false discovery rate (FDR)-corrected p-value. Nominal (uncorrected) correlations are presented for exploratory purposes. EDI, Eating Disorder Inventory; BMI, body mass index.

**Table S4**. Top 10 strongest Spearman correlations between differentially abundant taxa and clinical variables in the AN group.

| Taxon | Clinical variable | Spearman's ρ | *p*-value | FDR-adjusted *p*-value |
| --- | --- | --- | --- | --- |
| Faecalibacterium sp. | sexual abuse | 0.424 | 0.019 | 0.234 |
| [Eubacterium] oxidoreducens group sp. | BMI | -0.406 | 0.026 | 0.314 |
| Lachnospiraceae NC2004 group sp. | bulimia | -0.399 | 0.029 | 0.347 |
| Faecalibacterium sp. | emotional neglect | 0.394 | 0.031 | 0.373 |
| Lachnospiraceae NC2004 group sp. | interoceptive awareness | -0.370 | 0.044 | 0.267 |
| Unclassified Actinobacteriota | interoceptive awareness | -0.369 | 0.045 | 0.267 |
| Lachnospiraceae NC2004 group sp. | drive for thinness | 0.352 | 0.057 | 0.392 |
| Faecalibacterium sp. | ineffectiveness | 0.348 | 0.060 | 0.718 |
| Holdemania massiliensis | drive for thinness | -0.341 | 0.065 | 0.392 |
| Lachnospiraceae NC2004 group sp. | body dissatisfaction | 0.336 | 0.070 | 0.492 |

Note: Correlations were calculated using Spearman's rank correlation test on data from 30 patients with anorexia nervosa (AN). P-values were corrected for multiple testing using the Benjamini–Hochberg false discovery rate (FDR) method. None of the correlations remained statistically significant after FDR correction (FDR < 0.05). The table displays the 10 correlations with the largest absolute coefficients to illustrate potential trends. Taxa are labeled with their finest available taxonomic classification.
